# Genetic Polymorphisms in *CYP2E1*: Association with Schizophrenia Susceptibility and Risperidone Response in the Chinese Han Population

**DOI:** 10.1371/journal.pone.0034809

**Published:** 2012-05-11

**Authors:** Ran Huo, Kefu Tang, Zhiyun Wei, Lu Shen, Yuyu Xiong, Xi Wu, Jiamin Niu, Xia Han, Zhengan Tian, Lun Yang, Guoyin Feng, Lin He, Shengying Qin

**Affiliations:** 1 Bio-X Institutes, Key Laboratory for the Genetics of Developmental and Neuropsychiatric Disorders, Ministry of Education, Shanghai Jiao Tong University, Shanghai, People’s Republic of China; 2 Shanghai Genomepilot Institutes, Shanghai, People’s Republic of China; 3 Laiwu Hospital, Shandong, People’s Republic of China; 4 Shanghai International Travel Healthcare Center, Shanghai, People’s Republic of China; 5 Shanghai Institute of Mental Health, Shanghai, People’s Republic of China; 6 Institutes of Biomedical Sciences, Fudan University, Shanghai, People’s Republic of China; IPO, Inst Port Oncology, Portugal

## Abstract

**Background:**

CYP2E1 is a member of the cytochrome P450 superfamily, which is involved in the metabolism and activation of both endobiotics and xenobiotics. The genetic polymorphisms of *CYP2E1* gene (Chromosome 10q26.3, Accession Number NC_000010.10) are reported to be related to the development of several mental diseases and to be involved in the clinical efficacy of some psychiatric medications. We investigated the possible association of *CYP2E1* polymorphisms with susceptibility to schizophrenia in the Chinese Han Population as well as the relationship with response to risperidone in schizophrenia patients.

**Methods:**

In a case-control study, we identified 11 polymorphisms in the 5' flanking region of *CYP2E1* in 228 schizophrenia patients and 384 healthy controls of Chinese Han origin. From among the cases, we chose 130 patients who had undergone 8 weeks of risperidone monotherapy to examine the relationship between their response to risperidone and *CYP2E1* polymorphisms. Clinical efficacy was assessed using the Brief Psychiatric Rating Scale (BPRS).

**Results:**

Statistically significant differences in allele or genotype frequencies were found between cases and controls at rs8192766 (genotype p = 0.0048, permutation p = 0.0483) and rs2070673 (allele: p = 0.0018, permutation p = 0.0199, OR = 1.4528 95%CI = 1.1487–1.8374; genotype: p = 0.0020, permutation p = 0.0225). In addition, a GTCAC haplotype containing 5 SNPs (rs3813867, rs2031920, rs2031921, rs3813870 and rs2031922) was observed to be significantly associated with schizophrenia (p = 7.47E-12, permutation p<0.0001). However, no association was found between *CYP2E1* polymorphisms/haplotypes and risperidone response.

**Conclusions:**

Our results suggest that *CYP2E1* may be a potential risk gene for schizophrenia in the Chinese Han population. However, polymorphisms of the *CYP2E1* gene may not contribute significantly to individual differences in the therapeutic efficacy of risperidone. Further studies in larger groups are warranted to confirm our results.

## Introduction

Schizophrenia (OMIM: 181500) is a complex and severe mental disorder with a prevalence of approximately 1% in the population worldwide [1] and is characterized by psychotic manifestations, such as hallucinations, delusions and cognitive deficits [2]. Although family, twin, and adoption studies provide strong evidence that genetic factors play a crucial etiological role in the disease, with heritability estimated at up to 80%, key components and relevant pathways underlying the disease have still to be identified. Recent genome-wide association studies (GWAS) have provided new evidence for genetic predisposition by discovering hundreds of thousands of single nucleotide polymorphisms (SNPs) and copy number variations (CNVs) related to schizophrenia covering the whole human genome [3], [4], [5]. Current GWAS studies support the theory that particular loci are important in certain populations [6].

Cytochrome P450 enzymes (P450s) constitute a superfamily of hemeproteins which play a central role in the oxidative metabolism and biotransformation of a variety of endogenous and exogenous compounds [7]. Interindividual variability in the expression of *P450* genes contributes significantly to the difference in the disposition of numerous substrates, including the metabolites of steroids, environmental toxins and drugs [8]. CYP2E1, a key member of the P450 superfamily, is involved in the metabolism and bioactivation of a large number of low molecular weight compounds, many of which are industrial solvents (e.g. ethanol, acetone and chloroform), drugs (e.g. acetaminophen, isoniazid, chlorzoxazone, trimethadione and d-benzphetamine) and procarcinogens (e.g. benzene, N-nitrosodimethylaminen, and styrene) [9], [10], [11]. The *CYP2E1* gene (Chromosome 10q26.3, Accession Number NC_000010.10) presents several genetic polymorphisms which play an important role in interindividual variability in drug response, drug-drug interactions and in susceptibility to chemical-induced diseases [12], [13]. Among the known polymorphisms, the CYP2E1*1C and CYP2E1*1D allele contain repeat sequences in the 5′ flanking region which may act as negative regulatory elements [14]. The most extensively studied SNPs of *CYP2E1* are the PstI/RsaI site in the 5′ flanking region (CYP2E1*5B; rs3813867/rs2031920) and the DraI site in intron 6 (CYP2E1*6). The CYP2E1*5 allele has been associated with increased risk of developing antituberculosis drug-induced hepatitis and hyperglycemia-induced liver injury [15], [16]. Recent meta-analyses suggest that *CYP2E1 Pst*I/*Rsa*I and *Dra*I polymorphisms may affect susceptibility to many cancers such as lung cancer and head and neck cancer [17], [18]. On the other hand, CYP2E1 can catalyze reactions to yield reactive oxygen species (ROS) resulting from incomplete reduction of oxygen due to leakage of electrons through the electron transport chain [19]. A cluster analysis of transcriptional alterations showed that genes related to energy metabolism and oxidative stress differentiated almost 90% of schizophrenia patients from controls [20]. We therefore considered that *CYP2E1* might be involved in the onset or etiology of schizophrenia. However, apart from a case-control study of Japanese subjects in 1997 (sample size: 41 cases and 75 controls) with the result that no linkage of the *CYP2E1 c1/c2* polymorphism to schizophrenia has been found [21], there has been virtually no comprehensive study of association between genetic polymorphisms of *CYP2E1* gene and schizophrenia.

Risperidone is a benzisoxazole derivative which is widely used as one of the first-line atypical antipsychotics in treatment and maintenance therapy in schizophrenia and other psychotic diseases [22]. As a second-generation antipsychotic (SGA), risperidone has several advantages over typical antipsychotics, including having fewer extrapyramidal side effects (EPS), lower risk of tardive dyskinesia (TD), and improvement of both the positive and negative symptoms of schizophrenia [23], [24]. However, significant interindividual variations in response to risperidone with respect to both therapeutic effects and adverse side effects have been reported [25], [26]. Several genes in the drug transporters (e.g. ATP-binding cassette (ABC) transporters) and drug targets (e.g. dopaminergic and serotoninergic systems) have been discovered to be correlated with risperidone efficacy, such as *ABCB1* [26], *DRD2* [27], *DRD3* [28], *SLC6A4* [29], *HTR2A* [30] and *HTR2C* [28]. With respect to drug metabolic enzymes, the metabolism of risperidone to 9-hydroyrisperidone and other metabolites is mainly mediated by CYP2D6 and CYP3A4 [31], [32]. Due to their similar pharmacological characteristics, the combined effect of the plasma concentration of risperidone plus 9-hydroxyrisperidone, which is referred to as the ‘active moiety’, is usually measured for drug monitoring [33]. The relationships between *CYP2D6*/*CYP3A4* polymorphisms and risperidone metabolism have been tested but no correlation with clinical response was found [34], [35]. Since CYP2E1 metabolizes many low molecular weight compounds including several psychotropics, evidence suggesting that *CYP2E1* polymorphisms are related to risperidone response is insufficient.

Systematic screening of the *CYP2E1* gene in the Chinese Han population has revealed a total of 22 SNPs in the *CYP2E1* gene, of which the most frequent alleles are the wild-type allele *1 (49.5%) followed by *7 (33.9%), and the *5 allele (16.1%) [36]. We therefore chose to analyze CYP2E1*1C/*1D, a variable number tandem repeat in the 5' flanking region (5′-VNTR) and 10 SNPs (rs3813865, rs3813866, rs8192766, rs3813867, rs2031920, rs2031921, rs3813870, rs2031922, rs2070672 and rs2070673) with allele frequencies approximately higher than 20% in the promoter of the *CYP2E1* gene. Consequently, the purpose of this study was to examine: 1) the association between the *CYP2E1* gene polymorphisms and schizophrenia, 2) the relationship between *CYP2E1* gene polymorphisms and the clinical efficacy of risperidone treatment in schizophrenia patients.

## Materials and Methods

### Subjects

A total of 228 unrelated schizophrenic patients between 15 and 60 years of age were recruited from the Shanghai Mental Health Center (China). The controls consisted of 384 unrelated healthy individuals between 18 and 53 years of age. Clinical data were collected from 130 inpatients (45 males and 85 females with a mean age of onset of 30.3±10.3 years and a mean age of 36.3±11.2 years) among the 228 schizophrenics mentioned above. All the patients were chosen according to the following criteria:

- Meeting the Diagnostic and Statistical Manual of Mental Disorders-IV (DSM-IV) criteria [37] for schizophrenia;
- No physical complications or other psychiatric diseases such as alcoholism or other substance abuse;
- No history suggesting resistance to antipsychotics treatment;
- Had received no medication for 4 weeks;
- Had not previously been treated with atypical antipsychotics.

All subjects recruited for this study were Han Chinese in origin. Written informed consent was obtained from each participant including the guardians on the behalf of the minor participants. The study protocol was approved by the Shanghai Ethical Committee of Human Genetic Resources.

### Clinical Assessment

Before risperidone monotherapy treatment, all the patients had been medication-free for more than 4 weeks as a ‘washout’ period. All patients received risperidone at a daily dose ranging from 2 to 4 mg/day administered by the prescribing clinicians. The dosage was adjusted on the basis of individual tolerance. Patients’ compliance was closely monitored and confirmed by the nursing staff. During the medication period of 8 weeks, no other drugs were given except biperiden for moderate EPS, sennoside for constipation and flunitrazepam for acute insomnia. Clinical response was assessed on the basis of clinical improvement using the Brief Psychiatric Rating Scale (BPRS) [38]. BPRS ratings were conducted independently by two fully qualified psychiatrists who were blind to the genotype of each patient. Blood samples for the determination of risperidone and 9-hydroxyrisperidone concentrations were extracted at the end of the 8-week treatment, between 8∶00 and 9∶00 a.m., about 12 h after the bedtime dose. The plasma concentrations of risperidone and 9-hydroxyrisperidone were analyzed using high performance liquid chromatography (HPLC) [39]. Prolactin concentrations were assayed by radioimmunometric assay (RIA). Each patient was in a corresponding steady-state condition when all the concentrations were measured.

### Genotyping

Genomic DNA was extracted from venous blood using the QiaAmp® isolation system (Qiagen, Basel, Switzerland). The 5′-VNTR were genotyped by polymerase chain reaction (PCR) and electrophoresed on a 1% agarose gel. The SNPs were genotyped by direct DNA sequencing, using an ABI BigDye® Terminator Cycle Sequencing Kit (Applied Biosystems, CA, USA) on an ABI PRISM 3730 DNA sequencer (Applied Biosystems). Detailed information about primer sequences, PCR conditions and preparation of DNA for sequencing are available on request. All genotypes were performed by researchers who were blind to the clinical outcome of antipsychotic treatment. All observed DNA sequences of *CYP2E1* were deposited in NCBI Genbank and assigned with an accession number NC_000010.10.

### Statistical Analysis

Statistical analyses were carried out using the program SPSS for Windows, Version 18.0 (SPSS Inc., Chicago, IL, USA). The case-control study, pairwise linkage disequilibrium (LD) estimation and haplotype reconstruction were performed using SHEsis (http://analysis.bio-x.cn) [40]. We also used Haploview 4.2 [41] to estimate Hardy-Weinberg equilibrium (HWE) and LD. For accurate multiple testing correction, p values of the alleles and genotypes were permutated 100,000 times. Gender differences of demographic and clinical variables were tested using the Student’s t-test. Pearson’s correlation coefficient was calculated using GraphPad Prism 5 to examine the relationship between potential confounding factors (age, age of onset, baseline BPRS score, levels of active moiety, change of prolactin levels or metabolic ratio (risperidone/9-hydroxyrisperidone)) and clinical improvements in BPRS scores. Association tests between genotypes and percentage reduction in BPRS scores (and other clinical parameters) were performed using one-way analysis of variance (ANOVA). In the analysis of genetic effects on therapeutic efficacy, univariate analysis of variance (UNIANOVA) was carried out to adjust the results of the association test. Variables significantly correlated with the percentage change in BPRS scores were selected as covariates for the control of confounding effects in UNIANOVA. Post hoc power calculations with pre-established effect size, α error probability and sample size were carried out using the G*Power version 3.1.3 program [42]. All tests were two-tailed and p<0.05 was considered statistically significant.

## Results

### Association Study between the CYP2E1 Gene Polymorphisms and Schizophrenia

#### Single marker association analyses

To investigate the association of the polymorphisms in the CYP2E1 gene with schizophrenia, we detected allele and genotype frequencies of 10 SNPs in the promoter of CYP2E1 and 5′-VNTR in 228 schizophrenic patients and 384 healthy controls. The distribution of the SNPs was in HWE except for rs8192766 in cases (p = 0.0094<0.05), but the deviation disappeared after Bonferroni correction (corrected p = 0.094>0.05). The data for genotype and allele frequencies are listed in Table 1. As shown, significant differences in allele or genotype frequencies were observed between cases and controls at 5′-VNTR (allele: p = 0.0113, OR = 0.6868 95%CI = 0.5131–0.9193; genotype: p = 0.0063), rs8192766 (genotype: p = 0.0048) and rs2070673 (allele: p = 0.0018, OR = 1.4528 95%CI = 1.1487–1.8374; genotype: p = 0.0020). However, after 100000 permutations, the significant difference in 5′-VNTR (allele permutation p = 0.1062; genotype permutation p = 0.0626) disappeared. Thus, rs8192766 (genotype permutation p = 0.0483) and rs2070673 (allele permutation p = 0.0199, genotype permutation p = 0.0225) were identified as being associated with susceptibility to schizophrenia.

**Table 1:** Association analysis of 11 markers in the *CYP2E1* gene among schizophrenia patients and controls.

Marker	Sample	Genotype (freq.)			p value	Permutationp value	Allele(freq.)		p value	Permutationp value	OR (95% CI)
		6/6	6/8	8/8			6	8			
5′-VNTR	case	128(0.5664)	92(0.4071)	6(0.0265)	**0.0063**	0.0626	348(0.7699)	104(0.2301)	**0.0113**	0.1062	0.6868(0.5131–0.9193)
	control	253(0.6894)	103(0.2807)	11(0.0300)			609(0.8297)	125(0.1703)			
		GG	GC	CC			G	C			
rs3813865	case	152(0.6667)	72(0.3158)	4(0.0175)	0.4881	0.9960	376(0.8246)	80(0.1754)	0.3550	0.9646	0.8672(0.6411–1.1730)
	control	235(0.6386)	121(0.3288)	12(0.0326)			591(0.8030)	145(0.1970)			
		TT	TA	AA			T	A			
rs3813866	case	140(0.6140)	78(0.3421)	10(0.0439)	0.8953	1.0000	358(0.7851)	98(0.2149)	0.9445	1.0000	0.9900(0.7450–1.3154)
	control	222(0.6049)	131(0.3569)	14(0.0381)			575(0.7834)	159(0.2166)			
		TT	TG	GG			T	G			
**rs8192766**	case	73(0.3202)	129(0.5658)	26(0.1140)	**0.0048**	**0.0483**	275(0.6031)	181(0.3969)	0.3371	0.9561	1.1279(0.8821–1.4423)
	control	137(0.4190)	139(0.4251)	51(0.1560)			413(0.6315)	241(0.3685)			
		GG	GC	CC			G	C			
rs3813867	case	166(0.7281)	52(0.2281)	10(0.0439)	0.1253	0.6488	384(0.8421)	72(0.1579)	0.0713	0.4583	0.7525(0.5522–1.0255)
	control	241(0.6496)	112(0.3019)	18(0.0485)			594(0.8005)	148(0.1995)			
		CC	CT	TT			C	T			
rs2031920	case	128(0.5614)	85(0.3728)	15(0.0658)	0.2490	0.8894	341(0.7478)	115(0.2522)	0.0934	0.5493	0.7904(0.6003–1.0406)
	control	234(0.6273)	121(0.3244)	18(0.0483)			589(0.7895)	157(0.2105)			
		TT	TC	CC			T	C			
rs2031921	case	128(0.5614)	85(0.3728)	15(0.0658)	0.3727	0.9704	341(0.7478)	115(0.2522)	0.1789	0.7791	1.2062(0.9175–1.5857)
	control	231(0.6193)	121(0.3244)	21(0.0563)			583(0.7815)	163(0.2185)			
		AA	AG	GG			A	G			
rs3813870	case	150(0.6579)	74(0.3246)	4(0.0175)	0.3218	0.9468	374(0.8202)	82(0.1798)	0.6975	1.0000	1.0628(0.7817–1.4451)
	control	222(0.6607)	101(0.3006)	13(0.0387)			545(0.8110)	127(0.1890)			
		TT	TC	CC			T	C			
rs2031922	case	128(0.5614)	85(0.3728)	15(0.0658)	0.3857	0.9762	341(0.7478)	115(0.2522)	0.1771	0.7710	1.2101(0.9172–1.5965)
	control	214(0.6097)	121(0.3447)	16(0.0456)			549(0.7821)	153(0.2179)			
		AA	AG	GG			A	G			
rs2070672	case	161(0.7061)	64(0.2807)	3(0.0132)	0.1101	0.6047	386(0.8465)	70(0.1535)	0.1780	0.7763	1.2401(0.9064–1.6964)
	control	260(0.6771)	107(0.2786)	17(0.0443)			627(0.8164)	141(0.1836)			
		AA	AT	TT			A	T			
**rs2070673**	case	45(0.1974)	123(0.5395)	60(0.2632)	**0.0020**	**0.0225**	213(0.4671)	243(0.5329)	**0.0018**	**0.0199**	1.4528(1.1487–1.8374)
	control	60(0.1563)	169(0.4401)	155(0.4036)			289(0.3763)	479(0.6237)			

VNTR : Variable number tandem repeat. OR: Odds ratio; CI: confidence interval.

### Haplotype Association Analyses

**Figure 1:**
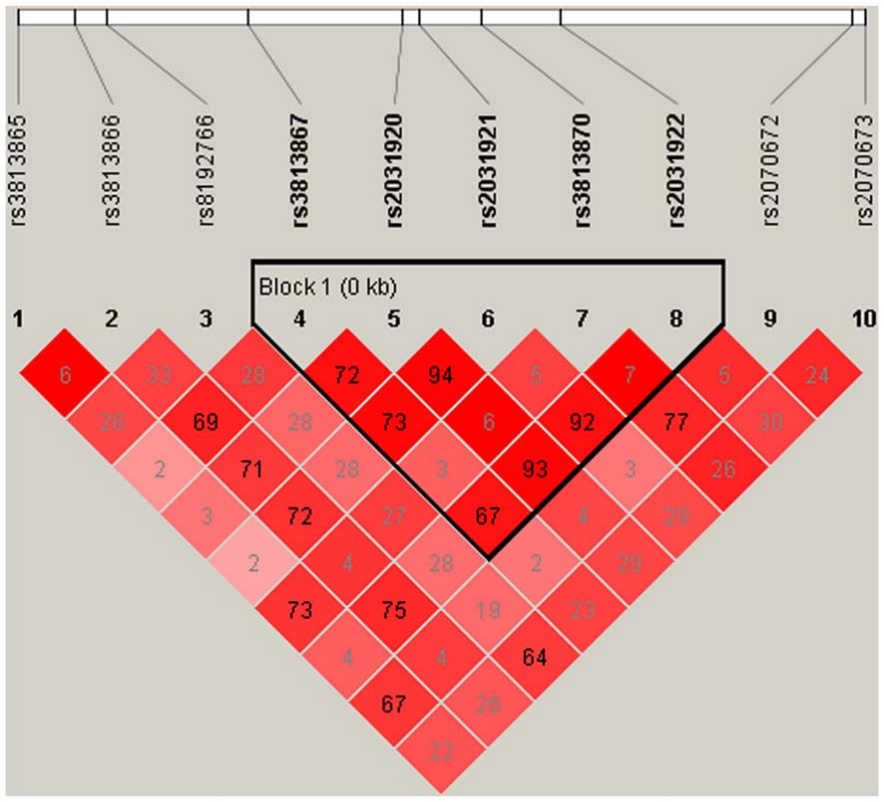
LD plot for *CYP2E1*. The LD plots were generated by Haploview 4.2. r^2^ between marker pairs is indicated by the shaded matrices. This figure shows that strong LD was observed between rs3813867, rs2031920, rs2031921, rs3813870 and rs2031922.

The estimation of LD for all pairs of SNP markers showed strong LD (r^2^>0.65) for rs3813867, rs2031920, rs2031921, rs3813870 and rs2031922 (Figure 1). Our results indicated that the 5 SNPs were in one LD block. After excluding the haplotypes with a frequency lower than 3% in patients and controls, only 4 haplotypes composed of the 5 SNPs were involved in the haplotype analysis. The overall haplotype frequency revealed significant differences between cases and controls (p = 1.19E-10). The GTCAC Haplotype which was more frequent in patients, proved to be significantly associated with schizophrenia (p = 7.47E-12, permutation p<0.0001) (Table 2).

**Table 2:** Estimated *CYP2E1* haplotype frequencies and p values among cases and controls.

Haplotype (rs3813867-rs2031920-rs2031921-rs3813870-rs2031922)	Case Frequency (%)	Control Frequency (%)	p value	Permutation p value	OR [95% CI]
C T C A C	15.5	19.3	0.0662	0.5671	0.742 [0.540–1.021]
G C T A T	56.6	58.7	0.2116	0.9955	0.856 [0.671–1.092]
G C T G T	18.0	18.1	0.7795	1.0000	0.957 [0.701–1.305]
G T C A C	9.7	0.9	7.47E-12	<0.0001	11.348 [4.811–26.767]
Global			1.19E-10	<0.0001	

Only haplotypes with a frequency higher than 3% are listed.

OR: Odds ratio; CI: confidence interval.

### Association Study between the CYP2E1 Gene Polymorphisms and Risperidone Response in Schizophrenia Patients

#### Clinical Profiles

All 130 patients treated with risperidone were available for assessment at the end of the 8-week treatment. Of the group, 67 were drug naive and the rest had prior exposure to conventional neuroleptics. 15 patients withdrew from the study because of concurrent somatic illness (n = 3), lack of efficacy (n = 6) or noncompliance (n = 6). Clinical profiles of the patients are displayed in Table 3. The patients were divided into two groups based on gender. Other than for baseline prolactin levels, there were no significant differences between sex groups in age, age of onset, steady-state plasma concentrations of risperidone and 9-hydroxyrisperidone, or in baseline BPRS scores on admission. This demonstrated that our sample data had no gender bias for primary clinical parameters. At the end of the 8-week treatment, 72 patients showed a reduction in BPRS scores >40% and were classified as responders and the other 58 patients were classified as non-responders. No significant differences in demographic characteristics or plasma levels of risperidone and 9-hydroxyrisperidone were found between the two groups (p>0.05) (data not shown).

**Table 3:** Clinical characteristics and baseline demographics of schizophrenia patients treated with risperidone.

Features	Total (n = 130)	Male (n = 45)	Female (n = 85)	p value
Age (years)	36.3±11.2	35.9±12.8	36.4±10.4	0.798
Age of onset (years)	30.3±10.3	28.4±10.6	31.3±10.1	0.132
Plasma risperidone levels (ng/ml)	7.6±6.3	7.0±6.4	7.9±6.3	0.469
Plasma 9-hydrorisperidone levels (ng/ml)	23.4±16.2	23.9±21.1	23.1±13.2	0.789
Baseline Prolactin (mIU/L)	775.2±733.8	508.0±340.2	907.2±835.5	0.004*
Baseline BPRS score	42.4±12.4	42.2±12.6	42.5±12.3	0.879

Data are shown as mean±SD. BPRS: Brief Psychiatric Rating Scale. *Significant difference (p<0.05) between males and females.

Using Pearson’s correlation analysis, we found that the baseline BPRS scores exerted a significant influence (r = 0.567, n = 130, p<0.001) on treatment efficacy as measured by the percentage reduction of BPRS scores, while no significant correlations between independent variables, such as age, age of onset, plasma active moiety, change of prolactin levels or metabolic ratio and the improvement of BPRS scores were found (Figure S1). Consequently, the baseline BPRS score was selected as a covariate to adjust for confounding effects in the analysis of association between *CYP2E1* genotypes and symptom improvement.

#### Effects of individual polymorphisms on clinical symptom improvement

All 11 polymorphisms were successfully genotyped in the 130 schizophrenic patients. For the 10 SNPs, no significant deviation from HWE was found in any of the subjects except for rs8192766 (p = 0.01<0.05). Table 4 shows the percentage change of BPRS score after the 8-week treatment among groups according to different genotypes. No significant association between percentage change of BPRS score and genotypes was observed before or after adjustment for the covariate effects (p>0.05). Moreover, there were no significant differences in allele or genotype frequencies in any of the 11 polymorphisms between the responder and non-responder groups (data not shown). These results suggest that CYP2E1 polymorphisms exhibit no significant effect on risperidone efficacy. Additionally, there were several significant discrepancies between genotypic subgroups of CYP2E1 polymorphisms in clinical parameters (p<0.05), such as active moiety which had positive association with rs3813866, rs3813867, rs2031920, rs2031921, rs2031922 and change of prolactin levels which had positive association with rs8192766 (Table S1).

**Table 4:** Comparison of reduction in BPRS score between genotype subgroups after the 8-week treatment.

				BPRS			
Marker	Genotype	Frequency (%)	p value for HWE	Baseline	Percentage reduction	p value	Adjusted p value
	6/6	73(56.1)		41.5±11.7	40.4±17.6		
5′-VNTR	6/8	53(40.8)	0.21	44.0±13.2	41.9±16.3	0.167	0.234
	8/8	4(3.1)		37.8±15.0	25.0±22.0		
	GG	86(66.2)		42.6±13.1	41.6±18.2		
rs3813865	GC	42(32.3)	0.33	41.9±11.0	39.3±14.7	0.322	0.156
	CC	2(1.5)		45.0±15.6	24.4±32.0		
	TT	74(56.9)		43.5±12.4	41.1±17.6		
rs3813866	TA	51(39.2)	0.40	40.2±10.3	39.0±17.2	0.496	0.540
	AA	5(3.9)		48.8±25.9	48.0±14.3		
	TT	37(28.5)		42.9±12.1	41.9±17.8		
rs8192766	TG	78(60.0)	0.01*	41.8±11.8	39.8±17.4	0.819	0.833
	GG	15(11.5)		44.3±16.0	41.3±16.3		
	GG	90(69.2)		42.6±11.7	40.5±17.0		
rs3813867	GC	35(26.9)	0.64	40.9±11.7	39.6±18.5	0.599	0.799
	CC	5(3.9)		48.8±25.9	48.0±14.3		
	CC	66(50.8)		43.5±12.4	40.9±18.0		
rs2031920	CT	57(43.8)	0.50	41.0±10.5	39.7±16.9	0.771	0.762
	TT	7(5.4)		43.1±23.4	44.6±14.5		
	TT	66(50.8)		43.5±12.4	40.9±18.0		
rs2031921	TC	57(43.8)	0.50	41.0±10.5	39.7±16.9	0.771	0.762
	CC	7(5.4)		43.1±23.4	44.6±14.5		
	AA	87(66.9)		41.9±12.9	40.9±18.0		
rs3813870	AG	41(31.6)	0.43	43.2±11.3	40.7±15.1	0.416	0.174
	GG	2(1.5)		45.0±15.6	24.4±32.0		
	TT	66(50.8)		43.5±12.4	40.9±18.0		
rs2031922	TC	57(43.8)	0.50	41.0±10.5	39.7±16.9	0.771	0.762
	CC	7(5.4)		43.1±23.4	44.6±14.5		
	AA	93(71.5)		41.9±12.9	40.7±18.0		
rs2070672	AG	36(27.7)	0.36	43.3±10.9	41.3±14.5	–	–
	GG	1(0.8)		–	–		
	AA	24(18.5)		40.7±14.1	39.1±18.2		
rs2070673	AT	77(59.2)	0.07	42.7±11.6	40.5±16.8	0.833	0.928
	TT	29(22.3)		43.0±13.1	42.0±18.2		

VNTR : Variable number tandem repeat; BPRS: Brief Psychiatric Rating Scale; HWE: Hardy-Weinberg equilibrium.

* Significant difference (p<0.05) showed deviation from HWE.

#### LD between SNPs and haplotype analysis

Pairwise LD between the 10 SNPs is shown in Table 5. Based on LD determinations, rs3813867, rs2031920, rs2031921, rs3813870 and rs2031922 were in moderate LD. These 5 SNPs with strong LD were included in the haplotype analysis. The 4 haplotypes with a frequency higher than 3% accounted for the majority of the haplotype diversity. The frequencies of the common haplotypes were compared between responders and non-responders. As shown in Table 6, there were no significant differences in haplotype distributions between the two groups (global χ^2^ = 0.574, p = 0.902).

**Table 5:** Pairwise polymorphisms LD analysis.

	rs3813865	rs3813866	rs8192766	rs3813867	rs2031920	rs2031921	rs3813870	rs2031922	rs2070672	rs2070673	
**rs3813865**		0.997	1.000	0.999	0.999	0.999	0.861	0.999	0.899	1.000	
**rs3813866**	0.066		1.000	1.000	0.952	0.952	0.999	0.952	0.998	0.909	
**rs8192766**	0.302	0.431		1.000	0.783	0.783	0.887	0.783	0.861	0.941	
**rs3813867**	0.045	0.683	0.295		0.966	0.966	0.998	0.966	0.995	0.872	
**rs2031920**	0.081	0.740	0.324	0.520		1.000	0.999	1.000	0.999	0.889	**D' value**
**rs2031921**	0.081	0.740	0.324	0.520	1.000		0.999	1.000	0.999	0.889	
**rs3813870**	0.722	0.064	0.232	0.044	0.078	0.078		0.999	0.967	1.000	
**rs2031922**	0.081	0.740	0.324	0.520	1.000	1.000	0.078		0.999	0.889	
**rs2070672**	0.644	0.052	0.179	0.035	0.064	0.064	0.765	0.064		1.000	
**rs2070673**	0.232	0.273	0.679	0.172	0.321	0.321	0.226	0.321	0.185		
					r^2^ value						

**Table 6:** Frequencies of estimated haplotypes and test statistics between responders and non-responders.

Haplotype (rs3813867-rs2031920-rs2031921-rs3813870-rs2031922)	Responders	Non-responders	χ^2^	p value	OR [95%CI]
C T C A C	0.181	0.154	0.292	0.589	1.199 [0.620–2.320]
G C T A T	0.542	0.559	0.135	0.713	0.912 [0.557–1.493]
G C T G T	0.180	0.164	0.101	0.751	1.111 [0.580–2.129]
G T C A C	0.097	0.114	0.209	0.647	0.831 [0.375–1.841]
Global			0.574	0.902	

Only haplotypes with a frequency higher than 3% are listed.

OR: Odds ratio; CI: confidence interval.

## Discussion

Our two-part study investigated the contribution of polymorphisms in the *CYP2E1* gene to both schizophrenia susceptibility and therapeutic response to risperidone in a Chinese Han population-based association analysis. We found statistically significant association for SNP rs8192766 and rs2070673 of the *CYP2E1* gene with schizophrenia. We also found that the haplotype consisting of rs3813867, rs2031920, rs2031921, rs3813870 and rs2031922 (GTCAC) was significantly associated with schizophrenia as shown by its overrepresentation in cases (OR = 11.348 95%CI = 4.811–26.767, p = 7.47E-12, permutation p<0.0001). Significantly, rs8192766 deviated from HWE, indicating possible inbreeding or population stratification. However, deviation in samples of affected individuals can also provide evidence for association [43]. While haplotype analysis revealed a significant association with schizophrenia, none of the single SNPs in this haplotype showed positive association. This may be due to the fact that haplotype analysis has a higher power than individual genetic markers in association analysis, given that haplotype analysis underlines the correlation between the individual markers. Our study has, for the first time, provided evidence for an association pattern of *CYP2E1* polymorphisms with schizophrenia in a Chinese Han population. However, no association of *CYP2E1* gene polymorphisms or related haplotypes with risperidone efficacy was found in the second part of the study. Post hoc power analysis revealed that the statistical power of the total sample size (228 cases VS 384 controls) in the case-control study was 0.95 as regards the association analysis with effect size d = 0.3, α = 0.05. For the pharmacogenomics study, the statistical power of the sample size (n = 130) in detecting significant association (p<0.05) was 0.87 with a medium effect size (d = 0.3). These results indicate that the sample size in our study was sufficient to achieve a considerably low risk of a type II error.

CYP2E1, which makes up only 1% of all the hepatic P450 isoforms, plays an important role in the metabolism of low molecular weight compounds and drugs. There is evidence to suggest that interindividual variability in the expression and functional activity of *CYP2E1* may be considerable. Genetic polymorphisms in *CYP2E1* have been linked to altered susceptibility to liver injury, head and neck cancer and other carcinomas in some epidemiological studies [16], [18]. There are many *CYP2E1* variant alleles but the functional significance of these variants is still unknown. None of the coding region variants consistently affects enzyme function, partially due to the fact that there are extremely rare polymorphisms in the coding regions of the *CYP2E1* gene. Therefore, any association study with human disease risk would require a high number of samples to generate enough statistical power. One novel nonsense mutation, which could result in the change of Arg to stop codon and a total loss of enzyme activity, has been detected for the first time in the Chinese population [36]. However, the mutation frequency presented only 0.5% and the effect on expression of the *CYP2E1* gene needs to be further investigated. Many studies have indicated that several upstream 5′ flanking mutations affect gene expression and response to inducers such as ethanol or obesity [13], [14]. Hence, our primary focus was on polymorphism in the 5′ flanking region that appear to influence gene expression.

CYP2E1, a potent producer of ROS, primarily expresses in the liver but also exists in extrahepatic tissues, including the brain [19], [44]. Induction of this enzyme in astrocytes causes oxidative stress, leading to increased production of metabolites of lipid peroxidation and decreased concentrations of glutathione [45], both of which are also seen in the brain in schizophrenia, Parkinson’s disease and Alzheimer’s disease [46]. Accordingly, we suspected that subjects with certain polymorphisms and haplotypes of *CYP2E1* might have an increased ability to activate endogenous or exogenous toxins, leading to increased oxidative stress in the brain and, therefore, an increased relative risk of schizophrenia. On the other hand, low CYP2E1 activity might protect individuals from schizophrenia because of the lower production of metabolites of ROS. There is also evidence to suggest that hyperactivity of dopaminergic neurons generates excess ROS in the acute stage of schizophrenia, which leads to subsequent neuronal damage and chronic disability [47]. Later, it was reported that aminochrome-dependent neurodegeneration of dopaminergic neurons in the mesolimbic system may contribute to the development of schizophrenia. Aminochrome, an oxidation metabolite of dopamine, is metabolized by NADPH-cytochrome P450 reductase to the highly neurotoxic o-semiquinone releasing large amounts of ROS. On the other hand, glutathione transferase M1b (GST-M1b) inhibits the formation of ROS during one-electron reduction of aminochrome catalyzed by NADPH-cytochrome P450 reductase [48]. Any defect in the mechanisms responsible for detoxification could lead to an excess of o-semiquinone and ROS in the dopaminergic, noradrenergic, or adrenergic nerve system, and finally to neurological disorder. Recent results also suggest a genetic effect of prolactin polymorphisms in prolactin levels indicating that independently of antipsychotic therapy subjects could have altered prolactin levels due to their genetic background [49]. In addition, prolactin can regulate oligodendrocyte precursor cells proliferation and promote intrinsic myelin repair, and may have potential for the treatment of neurological disorders [50]. Previous case-control studies aiming at the same cohort also have found some other candidate genes related to schizophrenia [51], [52]. Accordingly, it is possible that the combined effect of *CYP2E1* and other genes in related pathways might contribute to this complex disease. It is noteworthy that these schizophrenia susceptibility genes can be grouped into distinct families which reflect the processes disrupted in the schizophrenic brain and certain gene clusters can be linked together to form a defined signaling cascade involved in the *N*-methyl-D-aspartate (NMDA) receptor-dependent long-term potentiation and synaptic plasticity, that may be interconnected with oligodendrocyte and oxidative stress-related pathways [53]. Therefore, potential gene-gene interactions with respect to the schizophrenia phenotype measured in this cohort need further investigation. In addition, further accumulation of data on the role of the *CYP2E1* polymorphisms in the pathogenesis of schizophrenia and cellular or protein expression experiments should be conducted.

With regards to the pharmacogenomic association study of *CYP2E1*, 11 polymorphisms tested in the first case-control study were also investigated for their possible association with risperidone response in the 130 unrelated schizophrenic patients. Assessing the relationship between the plasma concentration of antipsychotics and therapeutic efficacy could be considerable. Therefore, we first investigated the correlation between plasma active moiety concentration and clinical effect in all 130 schizophrenia patients after 8 weeks of risperidone treatment. Although it has been reported that concentration of active moiety was associated with improvement of Positive and Negative Syndrome Scale (PANSS) [54], we found no significant relationship between plasma active moiety and percentage improvement of BPRS scores which accords with a study by Spina *et al* [55]. This further indicates that active moiety concentration may not be a suitable tool for predicting the treatment effect in schizophrenia patients. Analysis of the clinical characteristics of the patients in our study shows that the baseline of prolactin levels in females was significantly higher than in males, which is consistent with normal gender differences (Table 3). In addition, several other potential confounding factors, such as age, age of onset, baseline BPRS scores, change of prolactin levels and metabolic ratio were examined in the context of BPRS score improvements in order to eliminate non-genetic effects in the statistical analysis. Finally, significant correlation was only observed in baseline BPRS scores (Figure S1). Additionally, the significant differences between genotypic subgroups of *CYP2E1* polymorphisms in several clinical parameters (Table S1) are not relevant to the clinical efficacy of risperidone. For instance, the change of prolactin levels has positive association with rs8192766 but negative with the risperidone response, which could be interpreted by the previous finding that antipsychotic drugs modulate prolactin levels but prolactin is not a steady indicator of treatment outcome [56]. We therefore selected baseline BPRS scores as the covariant in adjusting ANOVA. Nevertheless, no association between any genetic markers and general symptom improvement was observed. Furthermore, we found no significant difference of allele, genotype or haplotype frequencies between responder and non-responder groups.

The current study attempted to confirm genetic contributors to the symptom variability in risperidone efficacy. Contrary to our expectation, the results indicated that the effect of variations in the *CYP2E1* gene on therapeutic efficacy of risperidone appeared weak or non-existent. However, further replication studies are warranted to identify whether or not *CYP2E1* genotypes are clinically related in the antipsychotic treatment, as the second part of the study had a relatively small sample number.

In conclusion, the case-control study revealed that *CYP2E1* polymorphisms were associated with susceptibility to schizophrenia in the Chinese Han population. On the other hand, the results in the pharmacogenomic study indicated that there is no positive association between *CYP2E1* polymorphisms and risperidone therapeutic effect. Since *CYP2E1* polymorphism frequencies show variability across different races and ethnicities, as do most other xenobiotic-metabolizing enzymes. The frequencies and alleles detected in Han population are similar to those in the Japanese and other Asian populations. However, the comparison with Europeans, Americans, and some other Caucasians showed significant differences. For instance, CYP2E1*7 showed a high frequency of 33.9% in Chinese Han population compared to 3.7% among German [36]. Therefore, replication of these findings in large independent samples within populations with different genetic background is essential to help confirm the results. However, data from our study may provide a basis for future investigations on the role which *CYP2E1* plays in the etiology of schizophrenia and risperidone treatment outcome.

## Supporting Information

Figure S1 **Correlations between potential confounding factors and the improvement of BPRS scores.**
(TIF)

Table S1 **Comparison of clinical parameters between genotypic subgroups of** ***CYP2E1*** **polymorphisms after the 8-week treatment.**
(DOC)
