# Supplementary figures and images for: Genetic Polymorphisms in CYP2E1: Association with Schizophrenia Susceptibility and Risperidone Response in the Chinese Han Population
Source: PLoS One. 2012 May 11;7(5):e34809. doi: 10.1371/journal.pone.0034809 (PMC3350493; doi:10.1371/journal.pone.0034809)

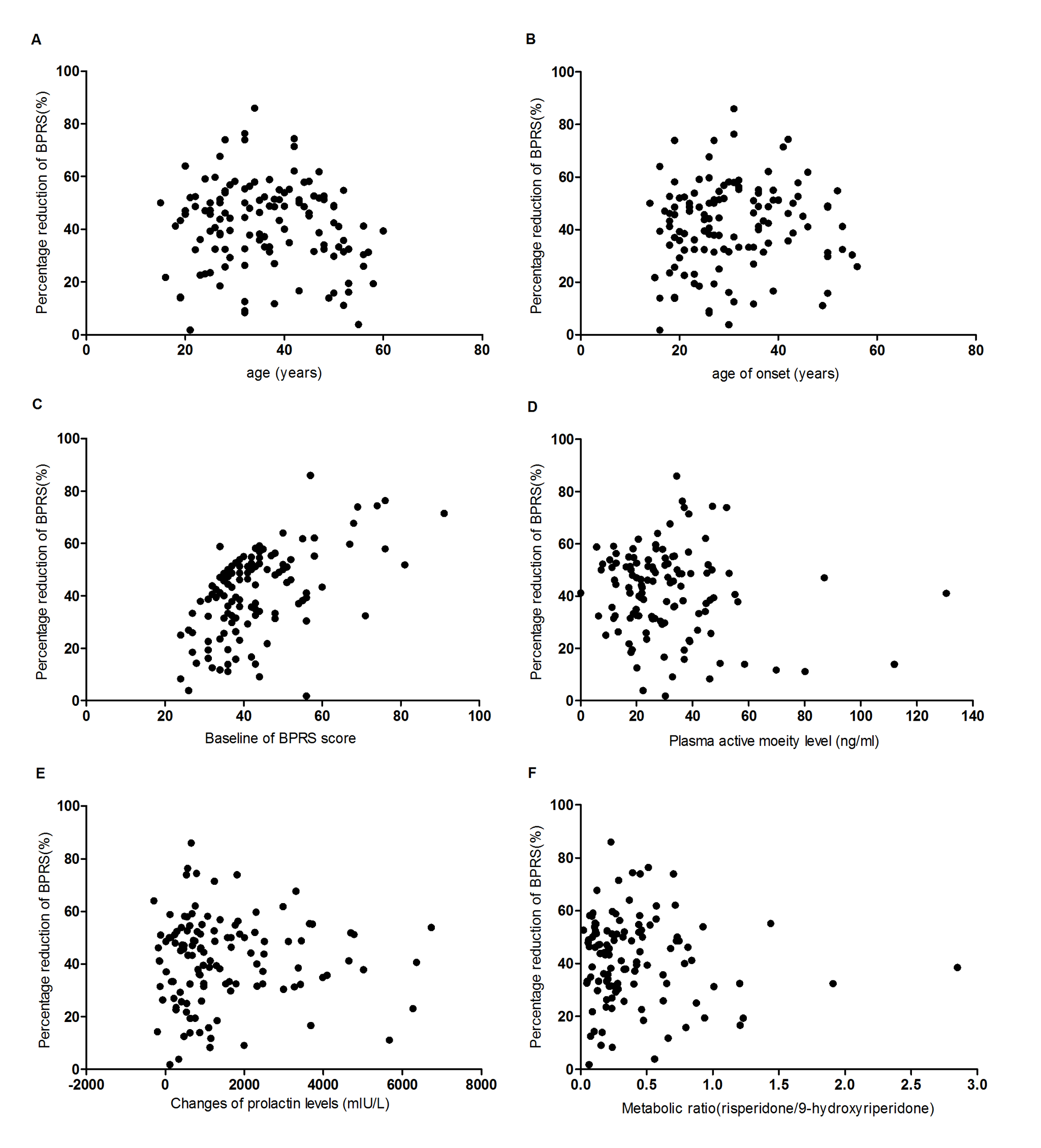

Supplement: Figure S1 — Correlations between potential confounding factors and the improvement of BPRS scores. (TIF) [file pone.0034809.s001.tif]
