# Supplementary material for: Genetic Polymorphisms in CYP2E1: Association with Schizophrenia Susceptibility and Risperidone Response in the Chinese Han Population
Source: PLoS One. 2012 May 11;7(5):e34809. doi: 10.1371/journal.pone.0034809 (PMC3350493; doi:10.1371/journal.pone.0034809)
Supplement: Table S1 — Comparison of clinical parameters between genotypic subgroups of CYP2E1 polymorphisms after the 8-week treatment. (DOC) [file pone.0034809.s002.doc]

**Table S1**. Comparison of clinical parameters between genotypic subgroups of *CYP2E1* polymorphisms after the 8-week treatment.

| Marker | Genotype | n | Active moiety | p value | n | Change of prolactin | p value | n | Metabolic ratio | p value |
| --- | --- | --- | --- | --- | --- | --- | --- | --- | --- | --- |
|  | 6/6 | 70 | 30.0±18.9 |  | 70 | 1467.0±1449.5 |  | 67 | 0.36±0.27 |  |
| 5’-VNTR | 6/8 | 50 | 31.4±18.9 | 0.610 | 51 | 1472.0±1625.1 | 0.563 | 47 | 0.46±0.53 | 0.358 |
|  | 8/8 | 4 | 21.9±10.3 |  | 4 | 644.8±495.3 |  | 4 | 0.48±0.33 |  |
|  | GG | 81 | 29.7±17.0 |  | 81 | 1471.3±1423.4 |  | 78 | 0.37±0.25 |  |
| rs3813865 | GC | 41 | 30.1±20.3 | 0.095 | 42 | 1442.3±1678.6 | 0.553 | 38 | 0.49±0.58 | 0.144 |
|  | CC | 2 | 58.7±40.1 |  | 2 | 292.4±255.1 |  | 2 | 0.10±0.05 |  |
|  | TT | 69 | 31.2±19.4 |  | 69 | 1371.7±1406.9 |  | 64 | 0.45±0.48 |  |
| rs3813866 | TA | 50 | 26.9±11.4 | 0.014 | 51 | 1577.1±1656.5 | 0.641 | 49 | 0.34±0.24 | 0.356 |
|  | AA | 5 | 51.5±44.8 |  | 5 | 1051.7±1281.1 |  | 5 | 0.43±0.27 |  |
|  | TT | 34 | 28.4±14.9 |  | 34 | 1329.8±1294.0 |  | 32 | 0.38±0.29 |  |
| rs8192766 | TG | 76 | 29.8±16.8 | 0.284 | 76 | 1654.9±1633.1 | 0.045 | 72 | 0.45±0.45 | 0.213 |
|  | GG | 14 | 37.6±32.2 |  | 15 | 623.7±893.7 |  | 14 | 0.25±0.22 |  |
|  | GG | 86 | 30.3±18.2 |  | 86 | 1439.4±1444.0 |  | 80 | 0.45±0.45 |  |
| rs3813867 | GC | 33 | 27.0±11.2 | 0.023 | 34 | 1508.7±1702.6 | 0.820 | 33 | 0.29±0.19 | 0.143 |
|  | CC | 5 | 51.5±44.8 |  | 5 | 1051.7±1281.1 |  | 5 | 0.43±0.27 |  |
|  | CC | 62 | 33.4±19.5 |  | 62 | 1357.1±1394.0 |  | 58 | 0.47±0.50 |  |
| rs2031920 | CT | 54 | 25.5±11.5 | 0.033 | 55 | 1613.7±1671.3 | 0.402 | 52 | 0.32±0.23 | 0.129 |
|  | TT | 8 | 38.5±38.5 |  | 8 | 930.8±989.4 |  | 8 | 0.44±0.28 |  |
|  | TT | 62 | 33.4±19.5 |  | 62 | 1357.1±1394.0 |  | 58 | 0.47±0.50 |  |
| rs2031921 | TC | 54 | 25.5±11.5 | 0.033 | 55 | 1613.7±1671.3 | 0.402 | 52 | 0.32±0.23 | 0.129 |
|  | CC | 8 | 38.5±38.5 |  | 8 | 930.8±989.4 |  | 8 | 0.44±0.28 |  |
|  | AA | 83 | 29.7±17.1 |  | 83 | 1422.6±1429.3 |  | 80 | 0.37±0.27 |  |
| rs3813870 | AG | 39 | 30.1±20.3 | 0.094 | 40 | 1542.0±1677.4 | 0.510 | 36 | 0.49±0.59 | 0.204 |
|  | GG | 2 | 58.7±40.1 |  | 2 | 292.4±255.1 |  | 2 | 0.10±0.05 |  |
|  | TT | 62 | 33.4±19.5 |  | 62 | 1357.1±1394.0 |  | 58 | 0.47±0.50 |  |
| rs2031922 | TC | 54 | 25.5±11.5 | 0.033 | 55 | 1613.7±1671.3 | 0.402 | 52 | 0.32±0.23 | 0.129 |
|  | CC | 8 | 38.5±38.5 |  | 8 | 930.8±989.4 |  | 8 | 0.44±0.28 |  |
|  | AA | 88 | 30.3±17.5 |  | 88 | 1496.9±1493.5 |  | 85 | 0.38±0.26 |  |
| rs2070672 | AG | 35 | 30.2±21.9 | — | 36 | 1347.3±1548.9 | — | 32 | 0.47±0.62 | — |
|  | GG | 1 | — |  | 1 | — |  | 1 | — |  |
|  | AA | 23 | 33.2±27.2 |  | 24 | 941.8±1525.6 |  | 23 | 0.34±0.26 |  |
| rs2070673 | AT | 75 | 28.8±16.7 | 0.535 | 75 | 1694.6±1540.1 | 0.06 | 70 | 0.43±0.45 | 0.646 |
|  | TT | 26 | 32.1±14.7 |  | 26 | 1178.5±1244.8 |  | 25 | 0.40±0.31 |  |

VNTR: Variable number tandem repeat; BPRS: Brief Psychiatric Rating Scale; Active Moiety: risperidone plus 9-hydroxyrisperidone. Metabolic ratio: risperidone/9-hydroxyrisperidone.
